# Impact of Pre-Analytical Time on the Recovery of Pathogens from Blood Cultures: Results from a Large Retrospective Survey

**DOI:** 10.1371/journal.pone.0169466

**Published:** 2017-01-03

**Authors:** Claudia Venturelli, Elena Righi, Lucia Borsari, Gabriella Aggazzotti, Stefano Busani, Cristina Mussini, Fabio Rumpianesi, Gian Maria Rossolini, Massimo Girardis

**Affiliations:** 1 Microbiology and Virology Laboratory, University Hospital of Modena, Modena, Italy; 2 Department of Biomedical, Metabolic and Neural Sciences, University of Modena and Reggio Emilia, Modena, Italy; 3 Anesthesia and Intensive Care Unit, University Hospital of Modena and University of Modena and Reggio Emilia, Modena, Italy; 4 Clinic of Infectious Diseases, University of Modena and Reggio Emilia, Modena, Italy; 5 Department of Experimental and Clinical Medicine, University of Firenze, Firenze, Italy; Universita degli Studi di Parma, ITALY

## Abstract

**Background:**

Prompt identification of bloodstream pathogens is essential for optimal management of patients. Significant changes in analytical methods have improved the turnaround time for laboratory diagnosis. Less attention has been paid to the time elapsing from blood collection to incubation and to its potential effect on recovery of pathogens. We evaluated the performance of blood cultures collected under typical hospital conditions in relation to the length of their pre-analytical time.

**Methods:**

We carried out a large retrospective study including 50,955 blood cultures collected, over a 30-month period, from 7,035 adult septic patients. Cultures were accepted by the laboratory only during opening time (Mon-Fri: 8am–4pm; Sat: 8am–2pm). Samples collected outside laboratory hours were stored at room temperature at clinical wards. All cultures were processed by automated culture systems. Day and time of blood collection and of culture incubation were known for all samples.

**Results:**

A maximum pre-analytical interval of 2 hours is recommended by guidelines. When the laboratory was open, 57% of cultures were processed within 2 h. When the laboratory was closed, 4.9% of cultures were processed within 2 h (*P*<0.001). Samples collected when the laboratory was closed showed pre-analytical times significantly longer than those collected when laboratory was open (median time: 13 h and 1 h, respectively, *P*<0.001). The prevalence of positive cultures was significantly lower for samples collected when the laboratory was closed compared to open (11% vs 13%, *P*<0.001). The probability of a positive result decreased of 16% when the laboratory was closed (OR:0.84; 95%CI:0.80–0.89, *P*<0.001). Further, each hour elapsed from blood collection to incubation resulted associated with a decrease of 0.3% (OR:0.997; 95%CI:0.994–0.999, *P*<0.001) in the probability of a positive result.

**Discussion:**

Delayed insertions of cultures into automated systems was associated with lower detection rates, with potentially important consequences for patients. In each hospital setting the logistic factors able to shorten pre-analytical time should be carefully investigated and specifically targeted.

## Introduction

Prompt identification of the infecting pathogen is essential for optimal management of patients with sepsis syndromes, and has been reported to significantly improve patient outcome, to reduce antibiotic resistance, to decrease healthcare costs, particularly with active antimicrobial stewardship involvement [1–6].

Collecting blood cultures prior to antimicrobial therapy remains the most important diagnostic tool for sepsis syndrome and it is recommended as a standard of care in all international guidelines for management of sepsis and septic shock [3, 6, 7]. The diagnosis of bloodstream infection is one of the most critical functions of clinical microbiology laboratories.

The development of automated blood culture monitoring systems and, more recently, of a number of molecular and mass spectrometry rapid identification methods have greatly shortened the turnaround time of the analytical phase [8–11]. In contrast, less attention has been paid to the several logistic factors able to influence the time elapsing from blood sampling to the start of blood culture processing in the laboratory (pre-analytical time), even though the length of this time has also shown to have an impact on the overall turnaround time and on the efficiency of the entire diagnostic process [5, 12–16].

Published guidelines recommend that the interval between the collection of blood and the entry of the bottles into an automated blood culture system should not be longer than 2 or 4 h; also manufacturer instructions indicate that inoculated vials should be transported to the laboratory as quickly as possible [17–19]. However, many laboratories do not provide a 24 h-service covering also holidays, and cultures obtained outside operating hours are often stored for longer times in clinical wards, usually at room temperature, before entering the incubators in the microbiological laboratory. In Italy, for instance, in a survey carried out in 2010 and involving about 100 laboratories located all over the country, it was observed that in the half of the investigated laboratories bottles were not immediately incubated during nightshifts [20]. Sometimes, the off-site location of the collection site causes long transport times, significantly increasing the pre-analytical overall time [16]. In Europe, according to a survey carried out across four countries (France, Germany, Italy and UK) in 2009 [21], most laboratories were closed overnight and only about 40% of them offered services during the weekend, with mean pre-analytical times of blood cultures ranging from 2 h in UK up to 20 h in German remote Laboratories. Long pre-analytical times due to transport times have been reported in the Netherlands as well, with 47% of cultures exceeding the recommended interval of 4 h [12].

Overall, studies on the recovery of microorganisms as a function of pre-analytical time are limited, and the actual conditions at which a reduction in sensitivity could occur are not clearly established. Few authors have evaluated experimentally the effects of the delayed incubation of blood culture bottles using samples spiked with microorganisms chosen among the most frequently isolated species. They found that delayed incubation increased the number of false negative cultures for different microorganisms [22, 23]. A prospective randomized and controlled trial [13] has shown that immediate incubation of blood cultures was able to significantly reduce the time to growth detection, identification and susceptibility testing but did not evaluate the impact on the detection sensitivity. More recently, a study carried out in England [24] has retrospectively evaluated blood culture yield over one year in a single critical care unit: these authors observed that blood cultures were less likely to be positive if collected at weekends.

To our best knowledge, no studies specifically aimed at evaluating the potential effects of pre-analytical time on the recovery of pathogens in routine clinical settings have been carried out up to now. Thus, we conducted a large retrospective study assessing, over a 30-month period and under typical hospital operative conditions, the impact of the length of pre-analytical time on the performance of blood cultures collected from adult patients admitted at the University Hospital of Modena (Italy).

## Materials and Methods

### Setting

The University Hospital of Modena is a 700-bed tertiary-care teaching hospital in Emilia-Romagna Region (Northern Italy) with medical, surgical, oncological, obstetric and paediatric facilities, intensive care unit and bone marrow and solid organ transplantation units. The on-site laboratory of clinical microbiology and virology receives blood culture specimens from all wards. The total number of blood culture bottles processed each year is about 15,000. During the study period (from January 2008 to June 2011) the laboratory opened on weekdays from 8 am to 4 pm and on Saturdays from 8 am to 2 pm, while on Sundays and on local or national holidays it was closed. When two holidays occurred consecutively, on the second day the laboratory was open (8 am–2 pm).

### Blood culture collection and processing

All blood cultures were performed per physician order as part of routine patient care. According to the hospital protocol and regional clinical practice guidelines, after accurate skin antisepsis, two or three blood sets were collected as soon as possible for each potential episode of bloodstream infection. Each set consisted of an aerobic and an anaerobic blood culture bottle and, according to the manufacturer’s instructions, for adult patients, each bottle was filled by a sterile needle with 8 to 10 ml of blood immediately after blood sampling. Bottles were transported to the laboratory by service personnel, since no automatic transportation system was established in this hospital. As the laboratory accepted the blood cultures only during opening time, blood cultures taken outside working hours were stored at room temperature in the clinical ward (21–25°C, all year around as an air conditioning system is operating in the whole hospital) until they were transported at room temperature to the laboratory. Upon receipt in the laboratory, the volume of blood inoculated into each bottle was assessed visually by comparison with known volume standards (10 ml).

Blood cultures were processed using the BACTEC system (Becton Dickinson). Each set (Bactec Plus Aerobic/F and Bactec Plus Anaerobic/F) was processed according to the manufacturer’s instructions: bottles were incubated until they flagged as positive or until 5 days elapsed. Bottles signalled as positive by an instrument were removed. A Gram stain and subcultures onto solid plate were performed. Subsequently, identification of microorganisms and antimicrobial susceptibility testing were carried out by manual and automated methods (Vitek, bioMerieux). Genetic sequencing technologies (Roche) were performed for some strains that were not identified by conventional methods.

Each positive result was designated as “contaminant,” or “true positive”. A blood culture was considered to be contaminated if one or more of the following organisms were isolated from only one set of blood culture: coagulase-negative staphylococci (CoNS), *Corynebacterium* species, *Micrococcus* species, *Propionibacterium* species and *Bacillus* species [17, 25, 26].

### Data collection

For each blood culture the following parameters were retrospectively extracted from the laboratory database and the Bactec system’s software: the vial code, the collection time (day, hour, minute) of each blood sample; the time (hour, minute) of bottle entry into the instrument (blood culture incubation time), the type of blood processed (peripheral or central catheter blood), the main demographic characteristics of patient (sex and date of birth), patient’s admission ward, and the microbiological result.

Only blood cultures collected from adult patients (>18 y/o) were considered eligible for the study. All cultures resulted negative or positive were included in the study.

Pre-analytical time was defined as the time difference between the collection time and the blood culture incubation time. Transport time and laboratory storage time before incubation are included in pre-analytical time.

### Statistical analyses

Length of pre-analytical time (median, interquartile range—IQR, 5^th^ and 95^th^ percentiles) was calculated for all blood cultures, and for cultures subgroups defined according to laboratory opening/closing time, day of sampling and results of blood culture. As data were not normally distributed, differences in length were compared using Mann-Whitney U test or Kruskal-Wallis test.

Frequencies of positive results for Bacteria, Yeasts and the main microbial subgroups were calculated for all blood cultures included in the study and for cultures subgroups defined by type of blood collected (peripheral or central catheter blood), collection time (during laboratory opening/closing time) and different categories of pre-analytical time lengths. Differences in frequency were tested by Pearson chi-squared test.

Multivariate logistic regression analyses were then performed in order to estimate the probability of positive results, as expressed as Odds Ratios (OR) and 95% Confidence Intervals (CI), according to laboratory opening/closing time or according to different pre-analytical time lengths. ORs were adjusted for demographic characteristics of subjects (sex and age), patient’s ward and for type of blood sampled (peripheral or central catheter blood) and were calculated both for overall positive results and for most frequently isolated microorganisms separately. For all statistical tests, a *P*<0.05 was considered to be statistically significant. All statistical tests were performed using IBM SPSS Statistics package ver. 21.

This study met the exemption criteria of clinical research of the ethics committee as it is an observational retrospective study based only on laboratory routinely collected data and did not allow the identification of patients.

## Results

A total of 52,548 blood culture bottles for diagnosis of bloodstream infections in adult patients were analysed over a 30-month period (January 2008 to June 2011). Among them, 1,437 blood cultures were excluded from the study as interpreted as contaminated. Further, 146 bottles were excluded as time of blood collection and/or of insertion in the automated monitoring system was missing. After all these exclusions, we evaluated 50,955 blood cultures (38,596–76% peripheral blood samples and 12,359–24% central catheter venepunctures) collected from 7,035 subjects (54.2% males; mean age: 64 ± 18 years) admitted in medical (44.4%), surgical (10.4%), onco-hematological (18.5%), intensive care (21.9%) and emergency (4.8%) wards.

Overall, 44,938 (88%) cultures resulted negative, 5,570 (11%) tested positively for Bacteria and 447 (1%) for Yeasts. As shown in Table 1, the most frequently isolated microorganism was *Escherichia coli* (22%), followed by CoNS (19%) and *Staphylococcus aureus* (14%). Among Yeasts, *Candida* gender accounted for 98% of isolates, with *Candida albicans* representing 54% of samples positive for Yeasts. In central catheter cultures the frequency of positive results was significantly higher than in peripheral blood samples (Bacteria: 14% vs 10%; Pearson chi-squared test, *P*<0.001; Yeasts: 1.5% vs 0.7%; Pearson chi-squared test, *P*<0.001).

**Table 1:** Microorganisms isolated in blood cultures.

Microorganism	n	%
**Gram-positive bacteria**		
Coagulase-negative staphylococci	1133	18.8
*Staphylococcus aureus*	859	14.3
*Enterococcus* spp.	506	8.4
*Streptococcus* spp. other than *S*. *pneumoniae*	222	3.7
*Streptococcus pneumoniae*	64	1.1
*Listeria monocytogenes*	19	0.3
Other	97	1.6
**Gram-negative bacteria**		
*Escherichia coli*	1325	22.0
Other Enterobacteriaceae	505	8.4
*Klebsiella* spp.	300	5.0
*Pseudomonas aeruginosa*	229	3.8
*Acinetobacter* spp.	103	1.7
*Haemophilus influenzae*, *Neisseriae meningitidis*	11	0.2
*Brucella* spp.	7	0.1
Other	63	1.0
**Anaerobic bacteria**		
*Bacterioides* spp.	32	0.5
Other	24	0.4
**Yeasts and Fungi**		
*Candida albicans*	239	4.0
*Candida parapsilosis* complex	75	1.2
*Candida glabrata*	59	1.0
*Candida tropicalis*	30	0.5
*Candida guillermondii*	16	0.3
*Candida krusei*	8	0.1
Other *Candida* spp.	11	0.2
*Fusarium* spp.	6	0.1
Other	3	0.05

The length of pre-analytical time showed a broad variability (median time: 4 h; interquartile range: 2–16 h, 95^th^ percentile: 35 h). 14,426 blood samples (28.3%) were processed by a 2 h-interval, 24,935 (48.9%) by a 4 h-interval and the remaining samples were incubated after longer intervals (26.4% after 4 h and within 16 h, 17.8% after 16 h and within 24 h, and 6.9% after 24 h). No difference in pre-analytical times among weekdays (Monday to Friday) was observed (Kruskal-Wallis test, *P* = n.s.). On the contrary, as expected, significant differences in the length of pre-analytical time were recorded between weekdays and weekend/holidays and in relation to laboratory opening/closing times, with the longest pre-analytical time observed for blood samples collected during the laboratory weekend closing time (Table 2). Accordingly, during laboratory opening and closing hours, blood cultures with an entry time shorter than 2 h were respectively 57% and 4.9% and those incubated by a 4h-interval were respectively 80.1% and 23.5% (Pearson chi-squared tests, *P*<0.001). Further, when blood was collected during weekends, most samples (81.6%) were characterized by an entry time longer than 16 h.

**Table 2:** Pre-analytical time distribution (h) according to day and/or time of sampling.

Day or time of sampling	N	median	IQR	*P*^b^
Weekdays	41367	3	1–14	<0.001
Weekend/holidays	9588	17	2–36	
Lab open^a^	22905	1	1–3	<0.001
Lab closed	28050	13	4–16	
Lab closed, weekdays	22110	10	3–15	<0.001
Lab closed, weekend	5940	25	17–39	

^a^ Mon-Fri: 8 am–4 pm; Sat: 8 am–2 pm;

^b^ length distributions of pre-analytical time according to day and/or time of sampling were compared using the nonparametric Mann-Whitney U test

The distribution of pre-analytical times showed a significant shift towards longer times in negative samples (median: 4 h; 95^th^ percentile: 35 h) in comparison to positive cultures (median: 4 h; 95^th^ percentile: 25 h, Mann-Whitney U test, *P* = 0.023). Further, the frequency of positive blood cultures varied significantly according to the time of blood collection. When samples were collected during laboratory opening hours 13.0% of cultures were positive, while when blood was sampled during laboratory closing times only 10.8% of cultures tested positively (Table 3). During laboratory closing time the overall probability of having a positive result showed a significant decrease of about 16% (adjusted OR:0.84; 95%CI:0.80–0.89). Differences in the prevalence of positive samples and in the probability of a positive result were highlighted as well when blood cultures positive for Bacteria (all), Gram positive or Gram negative bacteria and Yeasts were considered separately. Similar trends were observed for many microorganisms, including CoNS (adjusted OR:0.80; 95%CI:0.71–0.91, *P*<0.001), *S*. *aureus* (adjusted OR:0.76; 95%CI:0.66–0.87, *P*<0.001), Enterococci (adjusted OR:0.80; 95%CI:0.67–0.96, *P*<0.05), *E*. *coli* (adjusted OR:0.90; 95%CI:0.81–1.00, *P* = 0.052), *Klebsiella* spp. (adjusted OR:0.82; 95%CI:0.65–1.03, P = 0.083) and *Pseudomonas aeruginosa* (adjusted OR:0.22; 95%CI:0.63–1.07, P = 0.143).

**Table 3:** Frequency (n, %) and probability of a positive result (OR and 95%CI) according to laboratory opening and closing times.

Microbiological results of Blood cultures	Lab open^a^ n (%)	Lab closed n (%)	OR^b^ (95%CI)	*p*^b^
Negative	19927 (87.0%)	25011 (89.2%)	1	
Positive (all)	2978 (13.0%)	3039 (10.8%)	0.84 (0.80–0.89)	<0.001
Bacteria (all)	2751 (12.0%)	2819 (10.0%)	0.84 (0.80–0.89)	<0.001
Gram positive bacteria	1483 (6.9%)	1417 (5.4%)	0.80 (0.74–0.86)	<0.001
Gram negative bacteria	1194 (5.7%)	1331 (5.1%)	0.90 (0.83–098)	0.01
Yeasts	227 (1.0%)	220 (0.8%)	0.85 (0.70–1.03)	0.090

^a^ Mon-Fri: 8am-4pm; Sat: 8 am-2 pm

^b^ each category of positive results has been compared with the category of negative results (referent category) in a separate multivariate logistic regression analysis. ORs were adjusted for type of blood sample (peripheral vs central catheter blood), clinical ward (medical, surgical, onco-hematological, intensive care, emergency units) and sex and age of the patient.

Regardless of when blood samples were taken (during laboratory opening or closing hours), a delay in processing blood cultures longer than 2 h (occurring for 36,529 blood cultures– 71.7%) was associated with a reduction in the probability of having a positive result. This reduction was observed both when all positive results were included in the analysis (adjusted OR:0.92; 95%CI:0.86–0.97, *P*<0.01) and when cultures positive for all Bacteria (adjusted OR:0.91; 95%CI:0.86–0.97, *P*<0.01) or Gram-positive bacteria (adjusted OR:0.87; 95%CI:0.81–0.95, *P* = 0.01) were considered independently.

Finally, the overall probability of having a positive result significantly decreased by 0.3% (adjusted OR: 0.997 95%CI: 0.994–0.999, *P* = 0.011) each further hour elapsed from blood collection to blood culture incubation.

## Discussion

As far as we know, this was the first study retrospectively investigating the performance of blood cultures in typical hospital and laboratory operative conditions in relation to the length of their pre-analytical storage time. Almost 51,000 blood cultures processed over a 30-month period were included in the study. Blood samples collected during laboratory closing hours (nightshifts, weekends and public holidays) were characterized by a pre-analytical time significantly longer than samples collected during laboratory opening hours. Regardless of when blood samples were taken (during laboratory opening or closing times), pre-analytical time resulted as an independent factor able to influence the frequency of positive results: blood cultures with longer entry delays were more likely to show negative results than blood cultures processed quicker.

It has been experimentally estimated that 0.3 to 15.3% of bottles containing Bacteria or Fungi are flagged negative by blood culture systems [22, 23, 27–32]. The main parameters associated with a false-negative signal are the length of pre-analytical time, the temperature at which the bottles are stored and the type of microorganism involved [22, 23, 27, 30–32]. Lee et al. [22], for instance, using a simulated bacteraemia model, studied the growth dynamics of *S*. *aureus*, *E*. *coli* and *P*. *aeruginosa* and observed that, especially for *P*. *aeruginosa*, the number of false negative results were affected by changes in preincubation storage times and temperature with the highest false-negative rates occurring following a pre-incubation for 48 h at 25°C or for 24 h at 37°C. Sautter et al. [23] selected fifteen clinical isolates of ten different microorganisms, including both common clinical organisms and some challenge organisms more difficult to recover, and observed an increasing number of false-negative results, especially for *S*. *aureus*, *S*. *pneumoniae*, *E*. *coli* and *P*. *aeruginosa*, when bottles were held for more than 24 h at 4°C or at room temperature or for more than 12 h at 37°C. As reported also by Klaerner et al. [27], who reported a failure of the automated system to detect non fermentative Gram negative bacteria when bottles were pre-incubated at 36°C for 8 h, system fails to detect positive bottles may occur when bottles entering the incubation system contain microorganisms which have already reached the stationary phase.

In clinical operative settings some previous studies on pre-analytical conditions and length and their effects on the time occurring for a microbiological diagnosis have been performed [14–15, 33–35]. None of these investigations looked at microorganism recovery or at changes in the probability of positive results. All these studies demonstrated a significant effect of storage times, together with storage conditions, on the length of the total time for a diagnosis. Due to the start of growing in blood culture prior to incubation, a reduction in the time to a positive result in association with a delayed entry was observed after incubation for some microorganisms in some of these studies [14, 33], however the it never compensated the time period spend outside the incubator system and therefore did not result in a net time benefit. More recently, a study carried out by Morton et al. [23] evaluated blood culture performance as a surrogate for the quality of support service provision. The authors observed that at weekends blood cultures were less likely to test positively than during weekdays and they hypothesized that this could be a consequence of the reduced provision of support laboratory services causing delays and errors in culture incubation and processing.

Due to the operative conditions established in our hospital during the study period, the pre-analytical time of blood cultures could last up to 17 h during nightshifts and up to 48 h during laboratory weekend or holiday closing time, with more than 50% of blood samples collected during weekends characterized by delayed entry times longer than 24 h. Only during weekly laboratory opening hours most (but not all) blood samples could be incubated in accordance with the intervals suggested by international guidelines (57% and 80% of blood cultures were incubated respectively by 2 h and 4 h from the moment of the sampling). We believe that this is a very common situation: the opening times and days adopted by our laboratory during the study period were similar to those reported by many other laboratories in different hospital settings all-around Europe [20, 21]. Further, the pre-analytical times observed in our study are in agreement with the storage times reported by other authors for hospitals with laboratories characterized by limited operating hours or with off-site laboratories [12, 14, 16, 21].

In order to increase the performance of blood cultures, laboratory opening time/days could be increased and, during closing hours, whenever possible, laboratories could endeavour to make automated blood culture systems available outside the laboratory or at the point of care of critically ill patients so as to enable immediate incubation. Almost twenty years ago Riest et al. [36] had already demonstrated that time benefit of continuously monitoring of blood cultures in incubators is lost when the loading and the processing of blood cultures is discontinuous, and Bengtsson et al. [37] had reported a significant reduction in bacterial diagnosis time with the implementation of a 24 h, 7 day continuous loading of blood culture system. More recently, Kerremans et al. [13] carried out a prospective randomized controlled clinical trial assessing the impact of immediate incubation outside laboratory hours of blood cultures on turnaround times and antibiotic prescription practices. They found out that this procedure was able to accelerate antibiotic switching and, potentially, to improve patient outcomes, to decrease antibiotic use and to reduce management costs. Traditionally, in many settings, during nightshifts and weekends, pre-incubation at 37°C without growth monitoring is available as well. Both Koh et al. [34] and van der Velden et al. [35] demonstrated that this procedure enabled earlier final reports than storage at room temperature. However, as reported by several authors, pre-incubation at 37°C increases false-negative rate, especially if it lasts for long time [22, 23, 27–30, 32, 35]. Thus, some authors suggested that gram staining and subculture on arrival into the laboratory and a visual inspection of all pre-incubated bottles for any sign of bacterial growth prior to insertion into the automated systems should be considered when a pre-incubation longer than 12 h is performed [27–30, 35]. As recommended by several laboratory practices and reference guidelines, pre-incubation without growth monitoring for more than 12 h should be discouraged and room temperature should be considered as the most appropriate storage and transport temperature [17, 18, 26, 31].

Further, during laboratory opening time, blood culture bottles should be readily submitted to the laboratory. Previous studies have identified several logistic factors able to act as significant predictors of longer storage times, including clinical ward or laboratory off-site location, clinical speciality, time of sampling and number of transports per day [12, 14]. Both the clinical and financial benefits related to a more rapid bacterial identification have been already reported by several authors [1, 2, 5, 13, 34–35, 38]. The financial costs of implementing potentially effective interventions can be significantly different depending on the action chosen. Making continuously monitoring blood culture incubators available outside the laboratory during its closing time could be an intervention not expensive and easy to integrate into the existing procedures of a microbiological laboratory as suggested by Kerremans et al. [13]. On the other hand, increasing the opening time/days of the laboratory could be an action much more expensive and challenging without planning large centralized laboratory facilities. Pre-incubating blood cultures at 37°C during laboratory closing time could significantly increase both staff and supplies costs as well, if staining and subculture are planned, as also reported by van der Velden et al. [35]. In each hospital setting, different logistic and organizational factors should be carefully investigated in order to identify the most critical ones and to target the most cost-effective actions able to improve the local situation.

This study, of course, is characterized by some methodological limitations. It is a retrospective analysis based only on laboratory electronically stored data, and therefore, the correspondence between blood culture results and clinical status of patients could not be evaluated. Information on sex, age and admission ward of patients was available, however further unknown parameters might have affected microbiological results, including timing of venepuncture, skin antisepsis, blood volume, antibiotic treatment prior to sampling and patient comorbidities. Nevertheless, there is no reason to think that during nightshifts or weekends the actual frequency of patients with bacteraemia or the effects of parameters affecting blood culture results could be different from that expected during laboratory opening times. Finally, only blood cultures collected from adult patients were considered eligible for the study. Paediatric blood cultures, however, were excluded as evidence suggests that contamination occurs more frequently in this population, especially in young infants. Several reasons, including the reduced blood volume often cultured, the lower number of blood culture sets usually collected, the common use of exiting intravenous catheters for obtaining cultures instead of peripheral venepunctures, have been identified to explain these findings [25; 31]. Our data and results refer to typical operative conditions and therefore describe what was actually occurring in our hospital: we believe that this is an important strength of the study. Further, the long study period (30 months) and the large number of blood samples (about 51,000) and patients (7,035) investigated significantly increased the power to detect differences in the probability of blood culture positive results according to the length of pre-analytical time.

In conclusion, in our study we observed a relationship between the length of storage time at room temperature of blood cultures and the recovery of microorganisms. Delayed insertions into continuous monitoring systems were associated with the probability of lower detection rates for different microorganisms with potentially important consequences in the management of patients. We hope that these results could support microbiologists, clinicians and hospital managers in the identification and implementation of strategic targeted actions aimed at minimizing turnaround time and at increasing microbiological performance of blood cultures, the most important diagnostic tool for patients with sepsis syndromes.
